# The diagnostic yield of a first EEG in children with suspected epilepsy: A retrospective age-related comparison between awake and sleep recordings

**DOI:** 10.1016/j.cnp.2025.05.002

**Published:** 2025-06-08

**Authors:** Greta Gustafsson, Anders Broström, Eva Svanborg, Magnus Vrethem, Martin Ulander

**Affiliations:** a Department of Clinical Neurophysiology, Linköping University Hospital, Region Östergotland, SE-581 85 Linköping, Sweden; b Division on Neurobiology, Dept of Biomedical and Clinical Sciences, Faculty of Medicine, Linköping University SE-581 83 Linköping, Sweden; c Hälsohögskolan, Jönköping University, Jönköping, Gjuterigatan 5, SE-553 18 Jönköping, Sweden

**Keywords:** Sleep EEG, Melatonin, Epileptiform activity, Sleep deprivation

## Abstract

**Objectives:**

Previous research has indicated a low diagnostic yield of initial EEGs in epilepsy diagnosis in children. This study aimed at exploring factors affecting the diagnostic yield of a first EEG in different ages, and factors affecting the choice of EEG method.

**Methods:**

1097 EEGs performed as first recordings in children with suspected epilepsy were retrospectively analysed and divided into age groups: ≤ 5, 6–11 and 12–17 years. EEGs were classified as wake recordings with or without provocations, and sleep recordings: spontaneous sleep, sleep induced by deprivation or by melatonin premedication.

**Results:**

Epileptiform activity was found in 18 % of all patients, most common (30 %) in children 6–11 years old. It was more likely to be detected in melatonin-induced sleep EEGs compared to other types of sleep EEG. Presence of epileptiform discharges were positively associated to age, sleep, neurological and/or intellectual comorbidity in all children.

**Conclusions:**

The prevalence of epileptiform activity varies depending on the child́s age. Sleep EEG is advantageous as first recording, especially in very young children or with neurological comorbidity.

**Significance:**

Melatonin-induced sleep EEG has higher diagnostic yield compared to other types of sleep EEG. Age, seizure semiology and comorbidity affect the choice of EEG method.

## Highlights

• The prevalence of epileptiform activity varies depending on the age of the child.
• Sleep EEG is the preferable method as first recording in children.
• EEG after melatonin has higher diagnostic yield than other types of sleep EEGs.

## 1 Introduction

Electroencephalography (EEG) is a simple non-invasive investigation that is a cornerstone in the diagnostic work-up of epilepsy. However, it has been shown to have relatively low sensitivity as initial recording (Wirrell et al., 2010). The sensitivity increases with repeated EEGs and different provocation procedures such as photic stimulation, hyperventilation, and sleep deprivation (Kane et al., 2014, Marinig et al., 2000, Salinsky et al., 1987). EEG recordings can be challenging in young children, and it is easier to perform the EEG during sleep when movement artefacts can be avoided. It is also well-known that sleep, and not only sleep deprivation *per se*, can provoke epileptiform activity, especially in children (Kane et al., 2014, Marinig et al., 2000).

Traditionally, when suspicion of epilepsy arises, an initial EEG is performed during wakefulness, with or without provocations (Tatum et al., 2018). This shows the background activity which is crucial to assess the maturity of the child's brain development or the child's mental status in the event of absences. If the first EEG is negative, a second EEG is often performed during sleep (Carpay et al., 1997, Tatum et al., 2018). Physiological sleep is routinely used in very young children who habitually nap in the daytime (Carpay et al., 1997). Total, or partial sleep deprivation is used in clinical practice to get older children to sleep (Carpay et al., 1997, Shahar et al., 2010). This requires some effort on the part of the caregivers to keep the child awake before the EEG-investigation. Therefore, many laboratories have started to use melatonin to induce sleep for EEG recordings in children (Eisermann et al., 2010, Sander et al., 2012). EEG with melatonin-induced sleep does not require any prior preparation and can be performed in the same day as the referral. Melatonin does not affect epileptiform activity (Alix et al., 2018, Gustafsson et al., 2015).

However, previous research has paid little or no attention to the age of the children, even though the age could have great importance for the outcome (Baldin et al., 2014, Gilbert et al., 2004). There are two main reasons to assume this: firstly, the child’s ability to participate in the examination is greatly affected by age, and, secondly, different epilepsy syndromes, sensitive to different provocation methods, typically debut in different ages (Panayiotopoulos, 1999, Specchio et al., 2022, Wirrell et al., 2022).

The aim of this study was to explore factors affecting the diagnostic yield of the first EEG in children of different ages, as well as patient-related factors affecting the clinical choice of EEG method.

## 2 Methods

### 2.1 Study design

A retrospective population-based observational study.

### 2.2 Setting and participants

Children who had performed their first EEG in two public hospitals (Linköping University Hospital and Kalmar County Hospital) due to suspicion of epilepsy between 2009 and 2019 were included in the study. They were divided into three age groups: ≤5 years (i.e., 1 month to 5 years), 6–11 years and 12–17 years; and four different EEG recording types (wakefulness without provocations, wakefulness with provocations, sleep EEG with and without melatonin premedication). EEG recordings of children younger than 5 years who fell asleep spontaneously during EEG without provocations were categorised as sleep recordings. Children who performed sleep recordings without melatonin typically had prior partial sleep deprivation. Recordings were included backwards consecutively and stratified for age and type of EEG (i.e., starting from 2019 and counting back). All eligible children were included until either 100 children in a specific age bracket had undergone a specific type of EEG, or until the inclusion period was exhausted. Thus, the number of children in each stratum ranged from 28 to 100, for recruitment table see Appendix S1. Only the first EEG for each child was included in the study.

### 2.3 Variables and data source

All EEGs were analysed by physicians specialised in clinical neurophysiology. Data were collected from medical records concerning clinical symptoms/seizure type, underlying neurological diseases, presence of neurobehavioral conditions (e.g., autism spectrum disorders, attention deficit hyperactivity disorder), intellectual disability, anti-seizure medication, and time when EEG was performed in relation to seizure appearance. Seizure symptoms described in referrals were classified according to International League Against Epilepsy (ILAE) classification of seizure types 2017 and divided into seizures with generalized onset; motor and nonmotor (absence), focal seizures, and seizures with unknown or unclearly described symptoms (Fisher et al., 2017). Children with known neurological diseases (or syndromes) and with concomitant presence of neurobehavioral disorders and/or intellectual disability were classified as having neurological deficit. Data that were collected about the EEG recordings included method of EEG recording (i.e., wakefulness or sleep, type of sleep induction method, and type of provocations), occurrence of epileptiform discharges (including time from recording start to first appearance of epileptiform activity and whether it was during wakefulness, provocations, or sleep) and occurrence of sleep.

The study was approved by the Swedish Ethical Review Authority (Dnr2020-05220).

### 2.4 Statistical methods

All statistics were performed in R (The R Foundation for Statistical Computing, Vienna, Austria). Basic descriptive statistics are presented as absolute and relative frequencies and median + interquartile range (IQR). Univariate analyses of between-group differences were performed with chi-square test with Yates’ correction and Fisher’s exact test for categorical data. When more than two categories were compared, post-hoc Bonferroni correction was applied.

Regression was used for multivariate analyses. To explore factors affecting the diagnostic yield of first EEG in children of different ages (i.e., presence of epileptiform activity and sleep), logistic regression was employed. The choice of independent variables was made on theoretical assumptions as well as results from the univariate analyses. For each independent variable (i.e., presence of epileptiform activity and presence of sleep, respectively), four logistic regression models were calculated: one for all patients, and one for each age group (i.e., ≤ 5, 6–11 and 12–17 years). To assess what pre-examination factors affected the choice of EEG recording type (i.e., whether the EEG was performed as a wake or sleep recording and whether provocation methods were used or not), multinomial regression was performed with the type of EEG recording as the dependent variable and the clinical and demographic data known to the physician at referral time were used as independent variables.

## 3 Results

7 583 medical records were analysed. 1097 children who underwent their first EEG recording during 2009–2019 were included in the study. Demographic characteristics are shown in Table 1.

**Table 1:** Demographic characteristics of age groups.

Variable	All = 0–17 years	A = 0–5 years	B = 6–11 years	C = 12–17 years
Number, n (%)	1097 (100)	400 (36) *^b^	328(30)	369 (34)
Boys, n (%)	606 (55)	219(55)	204(62) *^c^	183(50)
Age, Median (IQR)	8 (10)	2(2)	9 (3)	15 (3)
Seizure type:				
Bilateral, n (%)	308 (28)	144 (36) *^b, c^	70 (21)	94 (25)
Focal, n (%)	288 (26)	112 (28)	94 (29)	82 (22)
Absence, n (%)	231 (21)	72 (18)	78 (24)	81 (22)
Unclear symptom, n (%)	270 (25)	72 (18)	86 (26) *^a^	112 (31) *^a^
Comorbidity:				
Neurological deficit, n (%)	130 (12)	38 (10)	50 (15)	42 (11)
Neurobehavioral deficit, n (%)	174 (16)	18 (5)	62(19) *^a^	94 (25) *^a^
Intellectual disability, n (%)	74 (7)	16 (4)	30 (9) *^a^	28 (8)
EEG within 72 h, n (%)	142 (13)	70 (21) *^b, c^	32 (11)	40 (12)
Antiepileptic treatment, n (%)	33 (3)	4 (1)	11 (3)	18 (5) *^a^

**Footnote:** Significant posthoc differences after Bonferroni correction: Significant value “*” = P < 0.05; Letters after the asterisk indicate between which groups there were significant differences in the post-hoc tests.

Missingness was generally low, 5–11 %, for comorbidities, without any significant differences between age groups or between employed methods. Only 3 % of the sample were using anti-seizure medication. This variable was not included in the presentation of further results due to the low number. EEG results are presented in Table 2, Table 3.

**Table 2:** EEG results by age.

Variable	All = 0–17 years	A = 0–5 years	B = 6–11 years	C = 12–17 years
N, (%)	1097 (100)	400 (36)	328 (30)	369 (34)
Normal EEG, n (%)	783 (71)	340 (85) *^b, c^	198 (60)	245 (66)
Epileptiform discharges:				
Total, n (%)	198 (18)	40 (10)	100 (30) *^a, c^	58 (16)
Bilateral, n (%)	38 (19)	9 (23)	18 (18)	11 (19)
Focal, n (%)	129 (66)	26 (65)	69 (69) *^a, c^	34 (59)
Multifocal, n (%)	31 (15)	5 (12)	13 (13)	13 (22)
Sleep occurrence, n (%)	481 (44)	197 (49) *^b^	130 (40)	154 (42)

**Footnote:** Significant posthoc differences after Bonferroni correction: Significant value “*” = P < 0.05; Letters after the asterisk indicate between which groups there were significant differences in the post-hoc tests.

**Table 3:** EEG results by age and employed EEG method.

Age	Variable	A = wake	B = wake+ Provocation	C = Sleep/sleep deprivation	D = Melatonin
0–17	Number, n (%)	300 (27) *^c^	300 (27) *^c^	221 (20)	276 (25) *^c^
	Sleep occurrence, n, (%)	27 (9)	32 (11)	190 (86)	232 (84)
	Epileptiform activity (EA):				
	Total, n (%)	81 (27) *^b, c^	23 (8)	34 (15) *^b^	60 (22) *^b^
	Bilateral, n (%)	17 (6)	0	5 (2)	16 (6)
	Focal, n (%)	48 (16) *^b^	19 (6)	24 (11)	38 (14) *^b^
	Multifocal, n (%)	16 (5)	4 (1)	5 (2)	6 (2)
0–5	Number, n (%)	100 (33)	100 (33)	100 (45)	100 (36)
	EA, n (%)	7 (7)	5 (5)	12 (12)	16 (16)
6–11	Number, n (%)	100 (33)	100 (33)	28 (13)	100 (36)
	EA, n (%)	45 (45) *^b^	15 (15)	9 (32)	31 (31)
12–17	Number, n (%)	100 (33)	100 (33)	93 (42)	76 (28)
	EA, n (%)	29 (29) *^b^	3 (3)	13 (14)	13 (17) *^b^

**Footnote: “**EA”- epileptiform activity. Significant posthoc differences after Bonferroni correction: Significant value “*” = P < 0.05; Letters after the asterisk indicate between which groups there were significant differences in the post-hoc tests.

### 3.1 Presence of sleep and epileptiform activity

10 % of children who performed wake EEG (with or without provocations) fell asleep spontaneously. When recording during sleep was intended, 85 % of the patients fell asleep. There were no differences in occurrence of sleep neither between wake EEG methods (EEG with vs. without provocations) or between sleep EEG methods (with vs. without melatonin) in any of the age categories. With increasing age and /or presence of neurobehavioral disorder, there were more difficulties in falling asleep (see Appendix S2).

In the recordings where epileptiform activity was found, the median time from recording start to the first occurrence of epileptiform activity was 2 min (Q1-Q3: 1–7 min), and 95 % had the first occurrence of epileptiform activity within 20 min. In the remaining 5 %, where epileptiform activity occurred after more than 20 min, it occurred within 25 min after sleep onset.

Epileptiform activity was found during resting wakefulness in 98 children, exclusively during provocation in 6 (2 photic stimulation and 4 hyperventilation), exclusively during sleep in 62 children and during both sleep and wakefulness in 32 children. However, in all children where epileptiform activity occurred only in wakefulness, sleep was never recorded. Thus, we do not know whether they might have had epileptiform activity also during sleep.

**Fig. 1:**
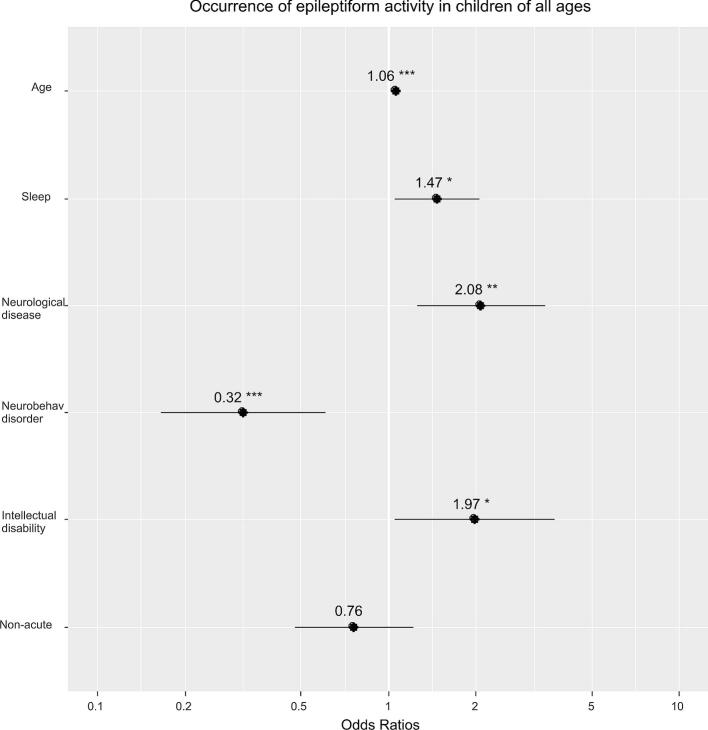
Factors associated to the presence of epileptiform activity in children 0–17 years old. Footnote: “Neurobehav”- neurobehavioral; “Non-acute”- non-acute EEG. ‘***’ – p < 0.001; ‘**’ −p < 0.01; ‘*’ – p < 0.05.

Fig. 1 and Appendix S3 show factors associated to the presence of epileptiform activity in children of all ages.

Similar results, as shown in Fig. 1, were even more pronounced in children ≤ 5 years old. In older children (6–17 years) presence of a neurobehavioral disorder was negatively associated to the occurrence of epileptiform activity in the EEG. In children 12–17 years of age, the likelihood of detecting epileptiform activity increased if the child underwent EEG within 72 h from referral or if the child had an underlying neurological disease (see Appendix S4).

Epileptiform activity was significantly (OR 1.81, p = 0.02) more likely to be detected in sleep EEG after melatonin premedication compared to other types of sleep EEG (spontaneous sleep or sleep deprivation). There was a trend towards a higher probability of epileptiform activity if the child had a neurological disease and /or intellectual disability (see Appendix S5).

### 3.2 Patient-related factors affecting the choice of EEG method

Demographic differences between the employed EEG methods are presented in Table 4. More detailed age-specific data are available in the supplements (see Appendix S6). Factors associated to the choice of EEG method according to univariate analyses and multinomial regression are presented in Appendix S6 and S7, respectively.

**Table 4:** Demographic differences between the employed EEG methods.

Variable	A = wake without provocation	B = wake with Provocation	C = Spontaneous sleep/ Sleep Deprivation	D = Melatonin
N, (%)	300 (27) *^c^	300 (27) *^c^	221 (20)	276 (25) *^c^
Boys, n (%)	173 (58)	163 (54)	113 (51)	157 (57)
Age, Median (IQR)	8 (10)	9 (10)	9 (14,1)	8 (9)
Seizure type:				
Bilateral, n (%)	89 (30)	62(21)	67 (30)	90 (33) *^b^
Focal, n (%)	80 (27) *^b^	49 (16)	68 (31) *^b^	91 (33) *^b^
Absence, n (%)	58 (19) *^c^	109 (36) *^a, c, d^	30 (14)	34 (12)
Unclear symptoms, n (%)	73 (24)	80 (27)	56 (25)	61 (22)
Comorbidity:				
Neurologic, n (%)	52 (17) *^b^	24 (8)	25 (11)	29 (11)
Neurobehavioral, n (%)	44 (15)	55 (18)	25 (11)	50 (18)
Intellectual disability, n (%)	32 (11)	17 (6)	11 (5)	14 (5)
EEG within 72 h, n (%)	92 (31) *^b, c, d^	13 (4)	33 (15) *^b, d^	4 (1)

**Footnote:** Significant posthoc differences after Bonferroni correction: Significant value *= P < 0.05; Letters after the asterisk indicate between which groups there were significant differences in the post-hoc tests.

Both univariate analyses and multinomial regression showed similar results: suspicion of absence epilepsy increased the probability to choose EEG with provocations and conversely this type of EEG was less likely to be performed if the child had focal seizures (Table 4) or comorbidity (neurological disease, intellectual disability (Appendix S7). If the EEG was performed within 72 h from referral, provocations and sleep recordings were less likely. The probability of choosing EEG with provocations or sleep deprivation increased with age compared to wake EEG without provocations (see Appendix S7).

## 4 Discussion

### 4.1 Factors affecting occurrence of epileptiform activity and sleep

Epileptiform activity was found in 18 % of all children, ranging from 10 % in ≤ 5 years old to 30 % in 6–11 years old children. Other studies have also showed variations in epileptiform discharges, ranging between 32–59 % in first EEGs in adults (Baldin et al., 2014) or 18–56 % in children (Wirrell, 2010). Some studies, however, include all abnormal EEG findings in the statistics (Hamiwka et al., 2008, Lizana et al., 2000). DeRoos et al. (2009) described higher number of normal EEGs in younger children, corroborating our findings. However, in the present study the prevalence of epileptiform activity is lower than that described in several other studies. The explanation could be that we examined a broadly selected sample, where all children with suspected epilepsy were included, also those where seizure symptoms were vaguely described.

Focal epileptiform discharges were the most common epileptiform finding contributing to 66 % of all epileptiform activity. It was especially common in the group 6–11-year-old-children where prevalence of epileptiform activity was 30 % which was significantly higher than in other age categories. In that age category focal seizure symptoms were also most often described in the referrals. This could be explained by the fact that the most common of the childhood epilepsies, namely self-limited epilepsy with centrotemporal spikes, debuts in that age group (Specchio et al., 2022).

Epileptiform activity in all children was correlated to age, sleep, neurological disease, and intellectual disability. This correlation was most significant in the youngest age category (≤5 years old). We found that the presence of neurobehavioral disorders was negatively correlated to the likelihood of epileptiform activity. This might seem surprising but could reflect diagnostic difficulties in distinguishing between epileptic seizures and paroxysmal behavioral changes in those children leading to a lower referral threshold for EEG examinations. In children 12–17 years of age, the likelihood of detecting epileptiform activity increased if the child underwent EEG within 72 h or if the child had underlying neurological disease. Several authors describe the importance of recording EEG shortly after seizures appear (Debicki, 2017, King et al., 1998). The correlation between time to EEG recording and epileptiform activity might be confounded by the fact that the children who had an EEG done within 72 h were more clinically affected.

Our finding that epileptiform activity was associated to sleep reproduces findings from previous research (Debicki, 2017, Gilbert et al., 2004). While certain types of epilepsy are more likely to produce epileptiform discharges and/or seizures during sleep, it has also been argued that the correlation of epileptiform activity and sleep is due to sleep EEGs being longer, thereby simply allowing for more time during which epileptiform activity can be recorded. We found, however, that epileptiform activity tends to occur early during the EEG (95 % had their first occurrence during the first 20 min), meaning that the duration of the EEG cannot explain the increased occurrence of epileptiform activity in sleep EEGs. This substantiates the recommendations from the IFCN regarding the recommended minimum duration of EEG for 20 min (Peltola et al., 2023). Burkholder et al. (2016) found, in a study of an unselected sample of adults and children, that late sleep onset was associated to having epileptiform activity after 30 min. This is in line with our own finding that children who had late epileptiform activity (i.e., >20 min), it occurred within 25 min after sleep onset. The clinical implication is that it is not the duration of the EEG that is of importance as much as the presence of sleep, and that sleep increases the diagnostic yield especially in children who have not had epileptiform activity during 20 min of recording in wakefulness.

When comparing different methods of sleep induction, epileptiform activity was more often found during melatonin induced sleep compared to spontaneous sleep or partial sleep deprivation. This is in a conflict with our and others previously reported results that melatonin does not affect the occurrence of epileptiform abnormalities in sleep EEG (Alix et al., 2018, Gustafsson et al., 2015, Sander et al., 2012). It can only be speculated that children who got melatonin had a shorter sleep latency, and therefore had longer sleep duration, but unfortunately, we cannot analyse this further as we have no data on sleep duration or sleep latency. We can at least state that melatonin is as effective as partial sleep deprivation to induce sleep and has several advantages compared to sleep deprivation. It can be used in the same day as EEG recording and can be especially useful in young children as both child and parent otherwise need to be awake during the night before sleep-deprived EEG.

### 4.2 Factors affecting the choice of EEG method

Age, seizure type and comorbidity had an impact on the choice of EEG method. With increasing age, the probability of choosing EEG with provocations increased. This might be due to the challenge of performing provocations in very young children, especially hyperventilation as it requires active participation from the child. Provocations during EEG were performed significantly more often in children with absences compared to those with other types of seizures. This is expected, as hyperventilation is considered the best provocative procedure to induce absence or typical epileptiform activity in childhood absence epilepsy (Kane et al., 2014, Panayiotopoulos, 1999). Therefore, it is important to perform provocations during EEG especially in those children who has suspicion for absence epilepsy. Provocations are easy to include in EEG recording, and in the interest of not having the child come back for repeated examinations which could delay treatment.

On the other hand, most children where focal seizures were described in referrals performed sleep EEG without provocations. Focal epileptiform activity that was most common in all EEGs has previously been described predominantly appears during sleep (Moore et al., 2021, Specchio et al., 2022). So, sleep EEG could possibly maximize the diagnostic yield of EEG in children with focal seizures. However, as clinical descriptions of seizures were vague in 25 % of the referrals, we opted not to explore this further in the present study.

In 35 % of the children, some type of comorbidity was registered; neurological, neurobehavioral, or intellectual disability. The prevalence of neurobehavioral conditions and intellectual disability increased significantly with age. This may be due to that the diagnosis of developmental disabilities and/or neurobehavioral deficits takes time. Furthermore, the less pronounced a neurobehavioral or intellectual dysfunction is, the later it is likely to be diagnosed. In children with underlying neurological disease and intellectual disability, EEG with provocations were less frequently performed as compared to other methods. To perform EEG in these children is often challenging and some authors consider performing EEG during sleep as preferable (Eisermann et al., 2010).

The timing for the EEG influences the choice of EEG method as 65 % of all acute EEGs were performed without provocations. This may have been chosen as it takes less time. Approximately half of the acute EEGs were done in children ≤5 years old. Those EEGs were performed as often during wakefulness as during sleep. This may be because when EEGs without provocations were performed, the children often fell asleep spontaneously and therefore the recordings were classified as sleep recordings. Seizure symptoms probably also prompted urgent examination.

The strength of our study is that we investigated a large sample of children in different ages where first EEGs were done using different EEG methods. There are a lot of studies that describes first EEGs, but usually in adults or in children as a whole age group or only using standard awake recordings (Debicki, 2017, Lizana et al., 2000, Wirrell and Significance, 2010). There are also several studies analysing sleep EEG recordings but usually as a second registration (Carpay et al., 1997, DeRoos et al., 2009, Shahar et al., 2010).

The main limitation of this study is that it is retrospective. This affects the quality of the collected data. Also, no causal inferences can be made as to how the clinical pictures of the individual cases influenced the type of EEGs that were performed.

It is not only the method of investigation itself that influences the result but also other factors such as physicians' request on how to conduct a particular investigation in different children. To be able to put results from studies with such limitations in a context, we also studied what factors affect the clinical decisions to perform a certain type of EEG recording. The routines of the EEG recordings in children differed between the two hospitals involved in this study. Nearly all children at Kalmar County Hospital performed sleep EEG after melatonin premedication. At Linköping University Hospital clinical routines gradually changed: during 2009–2010 most recordings were done in wakefulness, while during 2011–2019 most EEG were recorded during sleep. The methodology of sleep EEG recordings also changed from using partial sleep deprivation to melatonin premedication or age-dependent sleep deprivation for teenagers. These changes meant that children with similar clinical pictures would undergo different types of first EEGs due to factors unrelated to their symptoms. The choice of inclusion years was made to include children before and after these transitions in practice to reduce the effect of individualised decisions made by the diagnosing physicians.

In conclusion, correctly described data in referrals such as comorbidity and seizure symptoms have an impact on choice of recording method and therefore can influence the result of the EEG. The occurrence of epileptiform activity in children may vary, reflecting age-dependent prevalence of different epilepsy types. This knowledge can help to choose the appropriate EEG method and may increase the chances to detect epileptiform activity. Sleep EEG as a first recording in children has the advantage that the occurrence of epileptiform discharges covaries with sleep. Especially young children and children with neurological comorbidity can benefit from doing sleep EEG. Sleep EEG after melatonin premedication may be considered as this method is as good as or possibly better than sleep deprivation to detect epileptiform discharges.

## 5 Statement of Authorship

The study was conceptualized by Martin Ulander, Eva Svanborg, Magnus Vrethem and Anders Broström. Greta Gustafsson, Martin Ulander and Magnus Vrethem contributed to acquisition, analysis, and interpretation. The main author is Greta Gustafsson. All authors contributed to the revision and gave final approval of the manuscript.

## Funding

Financial support for the study was provided by Research Council of Southeast Sweden (FORSS) grant number: FORSS-911961.

## Declaration of competing interest

The authors declare that they have no known competing financial interests or personal relationships that could have appeared to influence the work reported in this paper.
