# Supplementary material for: The diagnostic yield of a first EEG in children with suspected epilepsy: A retrospective age-related comparison between awake and sleep recordings
Source: Clin Neurophysiol Pract. 2025 Jun 8;10:181–7. doi: 10.1016/j.cnp.2025.05.002 (PMC12210291; doi:10.1016/j.cnp.2025.05.002)
Supplement: Supplementary Data 1 [file mmc1.docx]

**S1.** Recruitment to the study.

|  | All children EEG 2019-2009 (n=7583) | | | | | | | | | | | |
| --- | --- | --- | --- | --- | --- | --- | --- | --- | --- | --- | --- | --- |
|  | Awake EEG without provocation (n=300) | | | Awake EEG with provocation  (n=300) | | | Sleep EEG (spontaneous/  PSD/ADSD) (n=221) | | | Sleep EEG after melatonin (n=276) | | |
| Age | 0-5 | 6-11 | 12-17 | 0-5 | 6-11 | 12-17 | 0-5 | 6-11 | 12-17 | 0-5 | 6-11 | 12-17 |
| N | 100 | 100 | 100 | 100 | 100 | 100 | 100 | 28 | 93 | 100 | 100 | 76 |
| Ye  ars* | 4 | 10 | 11 | 5 | 4 | 2 | 6 | 11 | 11 | 4 | 8 | 11 |

**Footnote:**

*- years needed to analyze for recruitment in a group.

PSD- partial sleep deprivation where children went to sleep as ordinary and wake up at 04.00 a.m.

ADSD- age-dependent sleep deprivation- children were asked to go to sleep 2 hours after their ordinary sleep time and wake up 2 hours before their ordinary wakeup time.

**S2**. Factors associated to ability to fall asleep in children in the total sample. Multivariate logistic regression.

| Factors | OR | OR 95%CI | p-value |
| --- | --- | --- | --- |
| Age | 0.89 | 0.84-0.94 | ˂0.001 *** |
| Gender | 0.89 | 0.52-1.52 | 0.67 |
| Neurological disease | 2.85 | 0.63-12.92 | 0.18 |
| Neurobehavioral disorder | 0.4 | 0.21-0.77 | ˂0.01 ** |
| Intellectual disability | 1.54 | 0.39-6.04 | 0.53 |
| Melatonin | 0.83 | 0.48-1.45 | 0.52 |

**Footnote:**

‘***’ – p <0.001; ‘**’ -p <0.01

**S3.** Factors associated to the presence of epileptiform activity in the total sample. Multivariate logistic regression.

| Factors | OR | OR95%CI | p-value |
| --- | --- | --- | --- |
| Age | 1.72 | 0.79–3.77 | ˂0.001 *** |
| Sleep | 1.47 | 1.05–2.05 | 0.02 * |
| Neurological disease | 2.08 | 1.25–3.44 | ˂0.01** |
| Neurobehavioral disorder | 0.32 | 0.17–0.61 | ˂0.001*** |
| Intellectual disability | 1.97 | 1.05–3.71 | 0.04 * |
| Non-acute EEG | 0.76 | 0.48 - 1.22 | 0.26 |

**Footnote:**

‘***’ – p <0.001; ‘**’ -p <0.01; ‘*’ – p <0.05

**S4.** Factors associated to the presence of epileptiform activity in children in different age groups (results from age-specific logistic regression models).

| Age groups | 0-5 years | | | 6-11 years | | | 12-17 years | | |
| --- | --- | --- | --- | --- | --- | --- | --- | --- | --- |
| Factors | OR | 95%CI | p-value | OR | 95%CI | p-value | OR | 95%CI | p-value |
| Age | 1.43 | 1.13-1.81 | ˂0.01 ** | 1.0 | 0.86 - 1.15 | 0.95 | 1.06 | 0.89–1.26 | 0.52 |
| Sleep | 2.88 | 1.3-6.37 | ˂0.01 ** | 1.3 | 0.78 - 2.16 | 0.31 | 1.7 | 0.88 - 3.25 | 0.11 |
| ND | 4.44 | 1.64-12 | ˂0.01 ** | 0.77 | 0.29 - 2.07 | 0.60 | 3.34 | 1.49 - 7.45 | ˂0.01 ** |
| NBD | N.A. | N.A. | N.A. | 0.28 | 0.11 - 0.70 | 0.01 ** | 0.30 | 0.11 - 0.83 | 0.02 * |
| ID | 4.41 | 1.1-17.64 | 0.04* | 1.74 | 0.59 - 5.15 | 0.32 | 2.19 | 0.77 - 6.20 | 0.14 |
| Non-acute | 1.02 | 0.37-2.83 | 0.97 | 0.84 | 0.37 - 1.89 | 0.67 | 0.31 | 0.13 - 0.75 | ˂0.01 ** |

**Footnote:**

ND- neurological disease; NBD- neurobehavioral disorder; ID- intellectual disability ‘**’ -p <0.01; ‘*’ – p <0.05. “N.A.”- not analyzed due to low prevalence.

**S5**. Factors associated to the presence of epileptiform activity in sleep EEG in children in the total sample. Multivariate logistic regression.

| Factors | OR | OR 95%CI | p-value |
| --- | --- | --- | --- |
| Age | 1.05 | 1.0 - 1.09 | 0.06 |
| Sleep | 1.72 | 0.79 - 3.77 | 0.17 |
| Neurological disease | 2.14 | 0.94 - 4.83 | 0.069 |
| Neurobehavioral disorder | 0.39 | 0.15 - 0.99 | 0.05 * |
| Intellectual disability | 2.59 | 0.96 - 7.02 | 0.06 |
| Non-acute EEG | 0.60 | 0.20 - 1.82 | 0.37 |
| Gender | 1.26 | 0.78 - 2.05 | 0.34 |
| Melatonin | 1.81 | 1.08 - 3.03 | 0.02 * |

**Footnote:**

‘*’ – p <0.05

**S6.** Demographic comparison between employed EEG methods.

| Age | Variable, n | A=Rest,  n=300 | B=Rest+  Provocation, n=300 | C=Sleep/  Sleep Deprivation, n=221 | D=  Melatonin,  N=276 |
| --- | --- | --- | --- | --- | --- |
| 0–5 | Number n (%) | 100 (33) | 100 (33) | 100 (45) | 100 (36) |
|  | Boys, n (%) | 58 (58) | 53 (53) | 56 (56) | 52 (52) |
|  | Median age (IQR) | 1 (2) | 3 (2) | 0,7 (0,8) | 3 (2) |
|  | Seizure type: Bilateral, n (%)  Focal, n (%)  Absence, n (%)  Unclear, n (%) | 35 (35)  30 (30)  20 (20)  15 (15) | 35 (35)  16 (16)  28 (28) *^c^  21 (21) | 27 (27)  36 (36) *^b^  11 (11)  26 (26) *^d^ | 47 (47) *^c^  30 (30)  13 (13)  10 (10) |
|  | Comorbidity:  ND, n (%)  NBD, n (%)  ID, n (%) | 6 (6)  2 (2)  3 (3) | 5 (5)  9 (9)  6 (6) | 17 (17) *^a, b^  1 (1)  5 (5) | 10 (10)  6 (6)  2 (2) |
|  | EEG within 72 hours, n (%) | 36 (36) *^b, d^ | 5 (5) | 28 (28) *^b, d^ | 1 (1) |
| 6–11 | Number n (%) | 100 (33) | 100 (33) | 28 (14) | 100 (36) |
|  | Boys, n (%) | 61 (61) | 68 (68) | 14 (50) | 61 (61) |
|  | Mean age (IQR) | 8 (3) | 9 (3) | 10 (3) | 8 (3) |
|  | Seizure type:  Bilateral, n (%)  Focal, n (%)  Absence, n (%)  Unclear, n (%) | 21 (21)  25 (25)  22 (22)  32 (32) | 16 (16)  19 (19)  37 (37) *^d^  28 (28) | 10 (36)  8 (29)  6 (21)  4 (14) | 23 (23)  42 (42) *^b^  13 (13)  22 (22) |
|  | Comorbidity:  ND, n (%)  NBD, n (%)  ID, n (%) | 22 (22) *^d^  21 (21)  17 (17) *^b, d^ | 15 (15)  15 (15)  5 (5) | 3 (11)  4 (14)  2 (7) | 10 (10)  22 (22)  6 (6) |
|  | EEG within 72 hours, n (%) | 23 (23) *^b, d^ | 5 (5) | 3 (11) *^d^ | 1 (1) |
| 12–17 | Number n (%) | 100 (33) | 100 (33) | 93 (42) | 76 (28) |
|  | Boys, n (%) | 54 (54) | 42 (42) | 43 (46) | 44 (58) |
|  | Mean age (IQR) | 15 (3) | 15 (3) | 15 (2) | 13 (4) |
|  | Seizure type:  Bilateral, n (%)  Focal, n (%)  Absence, n (%)  Unclear, n (%) | 33(33) *^b^  25(25)  16(16)  26(26) | 11 (11)  14 (14)  44 (44) *^a, c, d^  31 (31) | 30 (32) *^b^  24 (26)  13 (14)  26 (28) | 20 (26)  19 (25)  8 (11)  29 (38) |
|  | Comorbidity:  ND, n (%)  NBD, n (%)  ID, n (%) | 24 (24) *^b, c^  21 (21)  12 (12) | 4 (4)  31 (31)  6 (6) | 5 (5)  20 (22)  4 (4) | 9 (12)  22 (29)  6 (8) |
|  | EEG within 72 hours, n (%) | 33(33) *^b, c, d^ | 3 (3) | 2 (2) | 2 (3) |

**Footnote:**

ND- neurological disease; NBD- neurobehavioral disorder; ID- intellectual disability Significant posthoc differences: Significant value “***” =** P < 0.05; Letters after the asterisk indicate between which groups there were significant differences in the post-hoc tests.

**S7**. Patient-related factors affecting clinical choice of EEG method. Multinomial regression. Coefficients are calculated against baseline (wake EEG without provocations).

| Variable | Provocation  Coefficient ± Std Err (P value) | Sleep Deprivation  Coefficient ± Std Err (P value) | Melatonin  Coefficient ± Std Err (P value) |
| --- | --- | --- | --- |
| Gender | 0.044± 0.21  (0.84) | 0.082 ± 0.23  (0.72) | -0.004 ± 0.21 (0.98) |
| Age | 0.04± 0.02  (0.05) * | 0.06 ± 0.02  (0.005) ** | 0.004 ± 0.02  (0.85) |
| Focal seizure | -0.18± 0.27  (0.5) | 0.07 ± 0.26  (0.79) | 0.1 ± 0.24  (0.69) |
| Absence | 0.69 ± 0.26  (0.008) ** | -0.67 ± 0.31  (0.03) * | -0.87 ± 0.3  (0.003) ** |
| Neurological disease | -0.91 ± 0.41 7  (0.03) * | -0.98 ± 0.44  (0.02) * | -0.89 ± 0.4  (0.02) * |
| Neurobehavioral disorder | -0.02 ± 0.37  (0.95) | -0.24 ± 0.43  (0.57) | 0.14 ± 0.4  (0.73) |
| Antiepileptic drugs | -0.5 ± 0.69  (0.47) | -0.7 ± 0.73  (0.34) | -0.18 ± 0.6  (0.77) |
| Intellectual disability | -1.2 ± 0.5  (0.02) * | -0.69 ± 0.53  (0.19) | -0.72 ± 0.48  (0.14) |
| Non-acute EEG (>72 h) | 2.48 ± 0.4  (<0.001) *** | 1.84 ± 0.34  (<0.001) *** | 3.62 ± 0.6  (<0.001) *** |

**Footnote:**

‘***’ – p <0.001; ‘**’ -p <0.01; ‘*’ – p <0.05
